# Fruit Quality Analysis and Flavor Comprehensive Evaluation of Cherry Tomatoes of Different Colors

**DOI:** 10.3390/foods13121898

**Published:** 2024-06-17

**Authors:** Youlin Chang, Xiaodan Zhang, Cheng Wang, Ning Ma, Jianming Xie, Jing Zhang

**Affiliations:** College of Horticulture, Gansu Agricultural University, Lanzhou 730070, China; changyoulin98@163.com (Y.C.); zhangxiaod@gsau.edu.cn (X.Z.); gsauphd0810@outlook.com (C.W.); man@st.gsau.edu.cn (N.M.); xiejianming@gsau.edu.cn (J.X.)

**Keywords:** cherry tomato, quality, principal component analysis, cluster analysis

## Abstract

Cherry tomatoes are popular vegetables worldwide owing to their variety of colors and nutrients. However, an integrated evaluation of color and flavor has rarely been reported. This study examined the differences among red, brown, yellow, and green cherry tomatoes grown in the Jiuquan area. A comprehensive analysis of the flavor quality of these tomatoes, including sensory evaluation, electronic nose analysis, nutritional and flavor quality measurements, targeted metabolomics, and chemometrics, was conducted. Red tomatoes had the highest lycopene content, and green tomatoes had the highest soluble protein and vitamin C content. In cherry tomatoes, K is the most abundant macro element and Fe and Zn are the most abundant trace elements. Brown cherry tomatoes had significantly higher K, P, Mg, Cu and Fe contents than other colored tomatoes, and red tomatoes had significantly higher Zn content than other cherry tomatoes (218.8–724.3%). Yellow cherry tomatoes had the highest soluble sugar content, followed by red, brown and green tomatoes. A total of 20 amino acids of tomatoes were simultaneously determined by LC–MS. Yellow cherry tomatoes have the highest content of essential amino acids, aromatic amino acids and sweetness amino acids. Red tomatoes have the highest levels of non-essential and sourness amino acid contents. An analysis of 30 flavor indicators revealed that yellow tomatoes had the best flavor, followed by red, brown, and green tomatoes. Our work lays the foundation for future research on color and flavor formation in cherry tomatoes.

## 1. Introduction

Tomatoes (*Solanum lycopersicum* L.) are one of the most crucial commercial crops worldwide. They are rich in a range of flavonoids and phenolic acids which are considered to have potential antioxidant capacity [1,2]. Cherry tomatoes (*Solanum lycopersicum* var. *cerasiforme*), also known as sainfoin and pearl tomatoes, belong to the family Solanaceae, are one of the most popular varieties of tomatoes [3], and are thought to be the ancestor of the big-fruited tomato [4]. The cherry tomato contains high concentrations of sugars and acids [5], is consumed worldwide, and has been demonstrated to possess health-promoting properties because of its rich phytonutrient content [6].

Color is an important quality trait in tomatoes and is closely associated with their flavor, aroma, and nutritional components [7]. In fruit and vegetable crops, color is the main element of visual recognition for consumers [8], and the accumulation of pigments, such as carotenoids, is the main cause of color formation in red, orange, and yellow flowers and fruits [9]. All-trans-lycopene is a primary carotenoid that makes fruits mostly red [10], while anthocyanin pigments contribute to the purple color of fruit skins [11]. In addition, these pigments can affect the quality of fruits, are also considered to be bioactive compounds, and are thought to be associated with the prevention of cancer and cardiovascular disease in humans [12].

The characteristics that affect tomato quality are multifaceted, involve many processes at the plant and fruit levels, and depend on the interaction between field management, genetics and environmental factors [13]. The quality traits of cherry tomato fruits, including their appearance, nutrition and flavor, directly affect their commercial value [14], which is a key parameter evaluated in the context of commercial quality and food acceptability [15]. Cherry tomato fruits have a wide range of colors, such as the commonly found red variety, and the less studied pink, yellow, green and purple. Therefore, studying the nutritional and flavor qualities of tomatoes from different perspectives is important.

Over the last decade, many studies have helped elucidate the mechanism of color formation in differently colored tomatoes. For example, Lin et al. [7] discovered that the transcription factor MYB12, which regulates CHS (Chalcone Synthase) expression, has been shown to be the causative gene in a colorless skin *y* mutant tomato, which has pinkish fruits. Several genes associated with anthocyanin biosynthesis, including *AFT*, *ATV* and *ABG*, have been transferred into cultivated tomatoes, resulting in tomato lines with highly antioxidant purple fruits [16]. Most comprehensive evaluations of tomato quality haves focused on water-deficit irrigation [17], plant growth-promoting rhizobacteria [18], and climatic conditions [19]. However, an integrated evaluation of color and flavor has rarely been reported.

As stated previously, to determine the commercial and nutritional value of tomatoes [20], evaluating their quality from different perspectives is vital. As color is the most intuitive evaluation indicator, investigating the relationship between color, nutrition, and flavor is important for the selection of high-quality cherry tomatoes and the enhancement of their flavor qualities. In this study, classification models based on principal component analysis (PCA), cluster analysis, and correlation analysis were used to investigate the effect of different colors on the quality of tomato fruit and to provide a basis for subsequent research on the mechanisms of color and flavor formation in cherry tomatoes.

## 2. Materials and Methods

### 2.1. Plant Materials and Experimental Design

Four tomato varieties of different colors were selected for this study: (i) “red tomato, cv. ‘Sweet’ (RT)”; (ii) “brown tomato, cv. ‘Chocolate’ (CT)”; (iii) “yellow tomato, cv. ‘Song of Youth’ (YT)”; (iv) “green tomato, cv. ‘Romeo’ (GT)”. Cherry tomatoes were planted in the solar greenhouse of Gobi Agricultural Industrial Park, Suzhou District, Jiuquan City, from July to December 2021. The third ear of the ripe fruit from each plant was hand-picked and immediately transported to the laboratory. Tomato fruits of uniform size, free from pests, diseases and mechanical damage were selected to determine their appearance, nutritional value, and flavor quality. No fewer than 20 fruits of each color were selected and replicated three times. Some of the fruits were used to determine fruit diameter, hardness and weight per fruit. Other fruits were washed, homogenized, packed into cryopreservation tubes, treated with liquid nitrogen and frozen, then stored at −80 °C for the determination of relevant indicators.

### 2.2. Determination of Appearance Qualities

The length, horizontal diameter and vertical diameter of tomato fruits were measured by vernier calipers. The Fruit shape index was calculated from the horizontal and vertical diameters using the following equation:

Fruit shape index = vertical diameter/horizontal diameter(1)

The single fruit weight of the tomatoes was measured using an electronic balance. Firmness was determined by GY-4-J firmness tester (Top Cloud-Agri Technology Co., Ltd., Hangzhou, China). The skin color parameters of tomato fruits were measured using a colorimeter (CR-10 Plus; Konica Minolta, Inc., Tokyo, Japan), which provided the color surface coordinates L* for lightness, a* for the range between green and red, and b* for the range between blue and yellow.

### 2.3. Determination of Nutritional Qualities

The vitamin C content was determined using the 2,6-dichloroindophenol staining method [21]. Soluble protein content was determined by the Coomassie Brilliant Blue G-250 staining method [22]. Mineral elements were assayed using atomic absorption spectrophotometry [23]. Lycopene content was determined using the high-performance liquid chromatography (HPLC) method according to the China Chamber of Commerce for Import and Export of Medicines and Health Products (CCCHP) Group Standard Lycopene Plant Extracts (T/CCCMHPIE1.28-2018) [24], with slight modifications. Briefly, 5 g of tomato sample was placed in a 25-mL brown volumetric flask, to which 2.5 mL of dichloro-methane solution, with a concentration of 5 mg mL^−1^ BHT, was added. The sample was then fixed using dichloromethane and mixed using ultrasound. Subsequently, 1 mL of the sample was filtered through an organic 0.22-µm filter membrane and analyzed using a Waters HPLC system (Waters Technologies, Waters Corp., Milford, MA, USA) equipped with a 1525 pump and a 2998 photodiode array detector. An AC18 column (250 mm × 4.6 mm, 5 µm, Waters Symmetry) was used, and the mobile phase was methanol:dichloromethane = (92:8), the detection wavelength was 470 nm, the flow rate was 1.5 mL min^−1^, the column temperature was 30 °C, and the injection volume was 10 µL. The lycopene content in the sample was quantified by comparing the standard with the peak retention time and peak area of the sample.

### 2.4. Determination of Flavor Qualities

#### 2.4.1. Soluble Sugar, Titratable Acid Content and Organic Acid Content Assay

The salicylic acid sulfate method was used to determine soluble sugar content [25]. The titratable acid content of the tomato fruits was determined using the titration method [26]. Waters Acquity Arc HPLC was used to determine organic acid content (oxalic acid, tartaric acid, malic acid, α-ketoglutaric acid, citric acid, fumaric acid); this e system was conducted on an Atlantis T3 column (4.6 mm × 150 mm, 3 µm), The mobile phase was NaH_2_PO_4_ (20 mmol·L^−1^, pH 2.7), which was filtered through a 0.22 µm aqueous membrane and degassed via sonication before use; the flow rate for the mobile phase was 0.5 mL·min^−1^, the injection volume 20 µL, the detection wavelength was 210 nm, and the column temperature was maintained at 30 °C. Organic acid contents were expressed as g·kg^−1^ FW.

#### 2.4.2. Amino Acid Assay

Amino acid content was measured using the liquid chromatography–mass spectrometry (LC–MS) method according to Kong et al. [27]. Briefly, 100 mg of precisely weighed tomato lyophilized powder was mixed with 1 mL of 0.5 mol·L^−1^ aqueous hydrochloric acid solution in a centrifuge tube by vortexing for 5 min, and was then sonicated for 20 min at 25 °C in a water bath. After centrifugation at 8000 r·min^−1^ for 20 min, 250 μL of the supernatant was diluted to 1 mL with 80% acetonitrile aqueous solution, and passed through a 0.22 μm organic phase microporous membrane. The LC–MS system was equipped with an Agilent Infinity Lab Poroshell 120 HILIC-Z column (2.1 mm × 100 mm). The mobile phase A was 20 mmol·L^−1^ aqueous ammonium formate (pH = 3) and the mobile phase B was 20 mmol·L^−1^ aqueous ammonium formate (pH = 3) with 90% acetonitrile aqueous solution (*V*:*V* = 9:1); the flow rate was 0.5 mL min^−1^, the injection volume was 1 μL, ESI positive ionization mode was used, the desiccator temperature was 330 °C, the gas flow rate was 13.0 L·min^−1^, the intrathecal gas temperature was 390 °C, the sheath gas flow rate was 12.0 L·min^−1^, and the capillary voltage 1500 V. The amino acid profiles were analyzed according to the built-in UPLC–MS/MS database and quantified using the standard curve method.

#### 2.4.3. Phenolic Compound Assay

A 100 mg lyophilized sample of tomato was accurately weighed, and 2 mL of methanol was added to the sample. The phenolic compounds of the sample were extracted for 1 h at room temperature. The sample was then centrifuged at 8000 r min^−1^ for 10 min, and the resulting supernatant was passed through a 0.22 µm microporous membrane of organic phase for on-line determination. The chromatographic conditions were as follows: a HPLC C18 column (250 mm × 4.6 mm, 5 µm, Waters Symmetry) was used; mobile phases A and B were methanol and 1% acetic acid, respectively; a gradient elution at a flow rate of 1.1 mL min^−1^, the column temperature was 30 °C, and the injection volume was 10 μL. The detection of protocatechuic acid, chlorogenic acid, rutin, p-hydroxybenzoic acid and quercetin was carried out at 240 nm; gallic acid, cinnamic acid, benzoic acid, 4-coumaric acid, ferulic acid and naringenin were detected at 280 nm; gentianic acid, cynarin, erucic acid and kaempferol were detected at 322 nm. The characterization of individual phenolic compounds was carried out by comparing the retention time of the detected peaks and standards in the samples, and quantification was performed using the standard curve method.

### 2.5. Electronic Nose Analysis of the Volatile Content

The volatile components of the samples were analyzed using a portable electronic nose PEN3 (Airsense Analytics GmbH, Schwerin, Germany). The sensor array inside the PEN3 electronic nose consists of 10 chemical sensing elements used for identification. The signal response of the sensor is expressed as (G/G0), which is defined as the ratio of the electrical conductivity of volatile matter to that of pure air [28]. Different types of sensors can be used to detect different substances. The substance types and performance descriptions of the 10 sensors are presented in Table 1. The volatile components of tomatoes were analyzed according to the method described by Wei et al. [29], with slight modifications. Briefly, 8 g of tomato sample were weighed and ground with 1.5 g of anhydrous sodium sulfate and distilled water (2 mL). The sample was then transferred to a headspace bottle heated in a magnetic mixer at 70 °C for 15 min to equilibrate the internal headspace gas. The detection conditions were as follows: flushing for 60 s, a sensor zeroing time of 5 s, a pre-sampling time of 5 s, an injection flow rate of 400 mL·min^−1^, and a measurement time of 120 s. Three parallel samples were selected for each sensor to obtain stable maximum response values. A characteristic radar plot was then constructed.

### 2.6. Statistical Analysis

All experiments were performed in triplicate, and all results are expressed as mean ± standard error. Duncan’s multiple comparison test, analysis of variance, Pearson correlation analysis and PCA were performed using SPSS 22.0 (SPSS Institute Inc., Armonk, NY, USA). The significance level was set at *p* < 0.05 and *p* < 0.01. All figures were prepared using Origin Pro 2021 software (Origin Lab Inc., Northampton, MA, USA).

## 3. Results

### 3.1. Analysis of Appearance Qualities in Cherry Tomatoes of Different Fruit Colors

#### 3.1.1. Analysis of Skin Color Parameters

**Figure 1:**
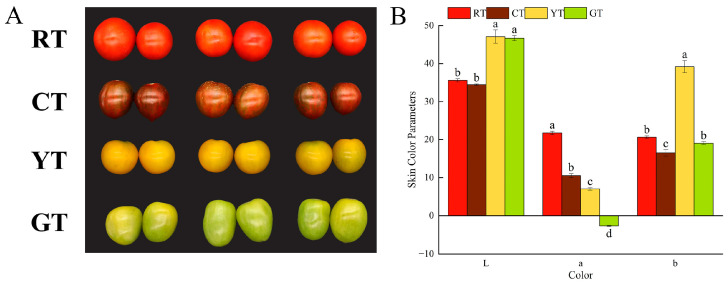
Skin color parameters of differently colored tomatoes. Fruit morphology (**A**); Skin color parameters (**B**). The vertical bars represent the mean ± standard error (SE) of three biological replicates. Different letters indicate significant differences according to Duncan’s multiple tests (*p* < 0.05). Abbreviations: CT, brown (chocolate) tomato; GT, green tomato; RT, red tomato; YT, yellow tomato.

Figure 1A shows the four different colors of cherry tomato sampled in this study. Skin color parameters are shown in Figure 1B. The color difference of tomatoes was compared by measuring the values of a*, b*, and L*. The L* value of YT and GT was significantly higher than that of other tomatoes. No significant difference was observed between RT and CT. The a* values of these tomatoes gradually decreased, with the highest values observed in RT, followed by CT, YT and GT. The GT a* value was the smallest, and negative. The b* value of the YT was significantly higher than that of the other tomatoes.

#### 3.1.2. Analysis of Morphological Indicators

Table 2 presents the morphological indicators of tomato fruits of various colors. The firmness of CT was significantly higher than that of the tomato fruits of other colors. The fruit shape indices of both RT and YT were close to 1, indicating that the fruits were approximately round. In addition, the fruit shape indices of CT and GT were higher than 1, and the fruits were very round. In some cases, no significant differences in firmness or fruit diameter was observed between the colors. The weight per fruit of differently colored tomatoes ranged from 20.53 to 31.16 g. RT had the highest single fruit weight, with 10.63 g more than the smallest GT.

### 3.2. Analysis of Nutritional Qualities in Cherry Tomatoes of Different Fruit Colors

#### 3.2.1. Analysis of Vitamin C, Soluble Protein and Lycopene Contents

**Figure 2:**
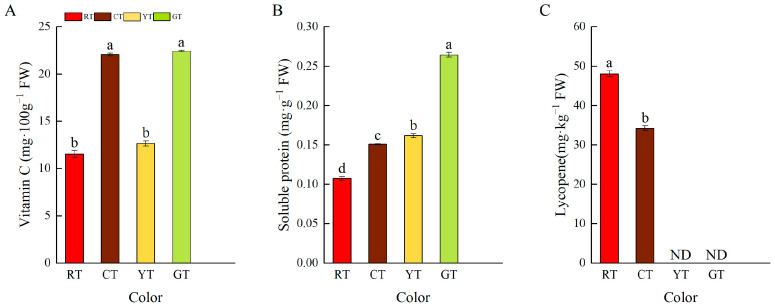
Nutritional quality of differently colored tomatoes. Vitamin C content (**A**); soluble protein content (**B**); lycopene content (**C**). Vertical bars represent the mean ± SE of three biological replicates. Different letters indicate significant differences according to Duncan’s multiple tests (*p* < 0.05). Abbreviations: ND, not detected.

As shown in Figure 2A, the vitamin C, soluble protein, and lycopene contents of the CT and GT groups were significantly higher than those of the other groups. RT had the lowest vitamin C content, with only 11.54 mg·100 g^−1^. Both CT and GT were rich in vitamin C. The soluble protein content of different colored tomatoes was shown in Figure 2B. Compared to other tomatoes, GT had the highest soluble protein content, which was 59.4% higher than that of RT. As shown in Figure 2C, lycopene was not detected in YT and GT. The lycopene content in RTs was significantly higher than that in CT. Briefly, lycopene content was correlated with tomato color formation. However, GT showed higher vitamin C and soluble protein contents than the other colored tomatoes.

#### 3.2.2. Analysis of Mineral Elements

**Figure 3:**
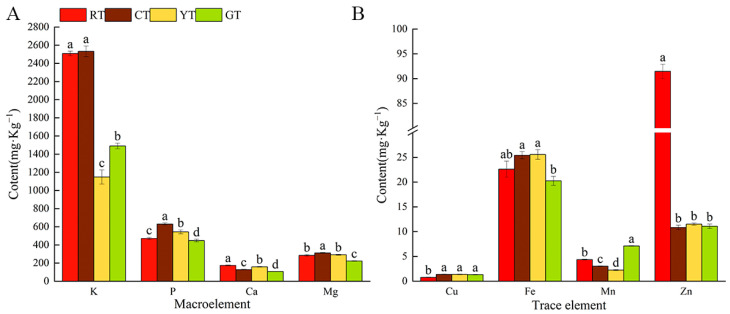
Mineral element contents of different color tomatoes. Macro element content (**A**), trace element content (**B**). The vertical bars represent the mean ± SE of three biological replicates. Different letters indicate significant differences according to Duncan’s multiple tests (*p* < 0.05).

The mineral element contents of each tomato variety are shown in Figure 3. In the case of macro elements, the K content was higher than that of the other elements for all colors. As for trace elements, higher levels of Fe and Zn were observed compared to the levels of Cu and Mn. The results also revealed that mineral element content varied between the different colors. RT contained high levels of K and Zn. CT contained high levels of K, P, Fe and Mg. YT contained high levels of Fe. GT contained high levels of Mn. No significant difference in Cu content was observed among the four colors. Finally, CT contained the most abundant mineral element.

#### 3.2.3. PCA and Cluster Analysis of Mineral Elements

**Figure 4:**
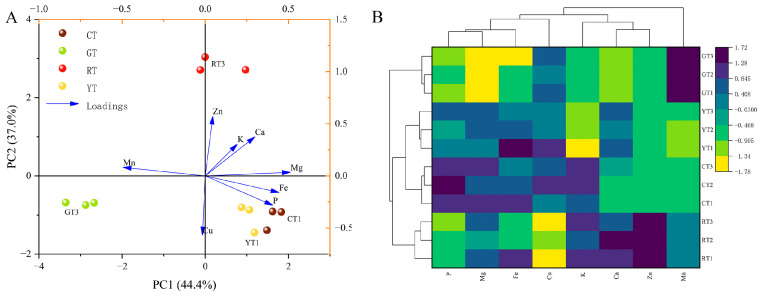
Principal component analysis (**A**) and cluster analysis (**B**) of mineral elements in tomato fruits. Abbreviations: PC1, principal component 1; PC2, principal component 2.

The PCA model for the mineral elements identified in cherry tomatoes of different colors is shown in Figure 4A. Two-dimensional PCA captured most of the changes in the mineral element of fruits; the sum of the first two principal components reached 81.4%, of which principal component 1 (PC1) and principal component 2 (PC2) accounted for 44.4% and 37.0% of the total variances, respectively. Furthermore, the loading plots demonstrated that Fe, Mg, and P had strong first principal component loadings, and Cu had strong second principal component loadings. The RT, CT, YT and GT groups were significantly separated according to PC1 and PC2. Furthermore, as shown in Figure 4B, the cluster heatmap analysis revealed that the differences in mineral element content between tomato groups were not significant. Finally, tomatoes with a color closer to blue had higher mineral contents. Briefly, the mineral content of the CT was richer than that of the other colored tomatoes.

### 3.3. Analysis of Flavor Qualities in Cherry Tomatoes of Different Fruit Colors

#### 3.3.1. Analysis of Amino Acids

Each amino acid accumulated inconsistently in cherry tomatoes of different colors. The results revealed that RT and YT contain high levels of amino acids. As shown in Table 3, the amino acids found at the highest levels in all the tomato groups were Lys, His, Glu and Asp. Compared to other colored tomatoes, the Thr, Lys, Trp, Phe, Leu, Ile, Met, Cys, Arg, Pro, Gly, Ala and Asn contents were the highest in YT, as were the total essential amino acid levels. Among the four tomato varieties, RT had the highest non-essential amino acids content.

Amino acids can influence flavor formation in tomato fruits, and different amino acids have different flavors, including bitterness, sweetness, and sourness. YT were significantly rich in total aromatic amino acids, bitter amino acids, and sweet amino acids. The contents of total sourness and umami amino acids were the second most abundant amino acids. We also observed that RT had the highest total amino acid contents. Overall, YT had the second highest total amino acid content, but the flavor amino acid content was significantly higher than that of the other colored tomatoes, suggesting that YT were more flavorful.

#### 3.3.2. PCA and Cluster Analysis of Amino Acids

**Figure 5:**
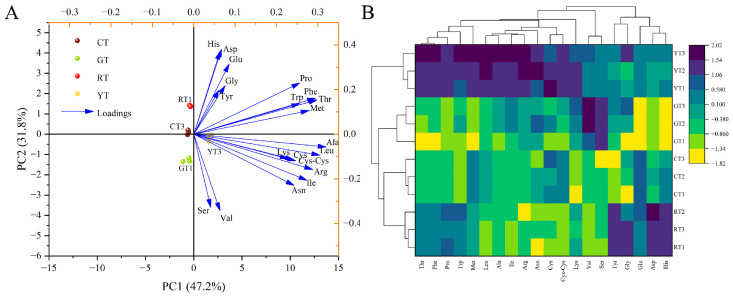
Principal component analysis (**A**) and cluster analysis (**B**) of amino acids of different color cherry tomatoes.

Amino acids are involved in the synthesis of flavor substances and enrich the taste of foods. A total of 20 different amino acids were detected in cherry tomatoes of different colors; a visual representation of a PCA-based amino acid classification model of different colored cherry tomatoes is shown in Figure 5A. We observed that the first and second principal components explained 79.0% of the total variance, with PC1 and PC2 accounting for 47.2% and 31.8%, respectively. The loading plots revealed that Thr, Phe and Leu were the main contributors to the first principal component. Moreover, His had strong second principal component loadings. Cherry tomatoes of different colors generated a clear separation based on PC1 and PC2. Both RT and CT were in the second quadrant, GT were in the third quadrant, and YT were in the fourth quadrant.

As shown in Figure 5B, the hierarchical cluster analysis was combined with the heat map analysis based on metabolite concentrations in all samples to simultaneously visualize clusters of samples and features. The results indicate that YT had a higher levels of amino acid metabolism. Notably, the color of the heat map correlates with the trends of the 20 amino acids in the sample. The group of deep blue represented higher levels of amino acids. The hierarchical cluster analysis successfully classified the different varieties based on amino acid metabolism and classified the four groups into three broad categories: YT, GT, CT and RT. This result is consistent with our PCA results. In conclusion, YT had the best amino acid profile, with high levels of the aromatic amino acids Phe and Trp.

#### 3.3.3. Analysis of Soluble Sugar, Titratable Acid, Soluble Solids and Organic Acid Contents

**Figure 6:**
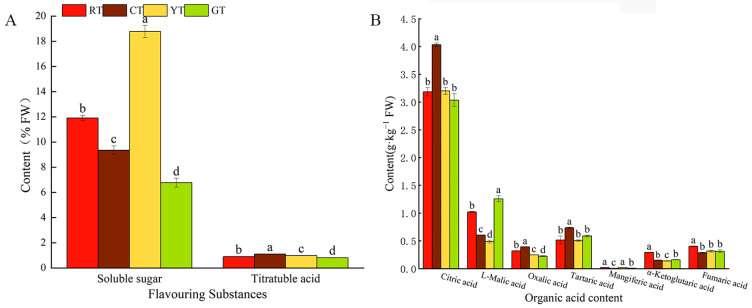
Flavoring substances and organic acid contents of differently colored cherry tomatoes. Flavoring substances (**A**), organic acid contents (**B**). The vertical bars represent the mean ± SE of three biological replicates. Different letters indicate significant differences according to Duncan’s multiple tests (*p* < 0.05).

As shown in Figure 6, the soluble sugar content was significantly different among the differently colored cherry tomatoes, and YT showed the highest content of soluble sugar. Similar RT and PT values were observed. Titratable acid affects the taste of tomato fruits; titratable acid levels were significantly higher in CT than in the other fruits, decreasing by 27.2% and 35.2% in RT and YT, respectively, compared with CT. Organic acids are important for fruit quality in terms of taste [30], and citric acid is the predominant organic acid in tomatoes; the citric acid content in CT was significantly higher than that in RT, YT and GT. Malic acid is another major organic acid in tomato fruits, and its content in YTs was 61.1% lower than that found in GTs. Minor levels of oxalic acid, tartaric acid, α-ketoglutaric, and fumaric acid, which varied from 0.14 to 0.59 g·kg^−1^, were detected. Meanwhile, YTs exhibited the lowest organic acid content among all tomatoes.

#### 3.3.4. Analysis of Phenolic Compounds

The phenolic compounds of cherry tomatoes of different colors is shown in Table 4. A total of 11 phenolic compounds were detected, including 4 flavonoids and 7 phenolic acids. The level of total phenolic acid compounds was significantly higher in YT than in the other colored cherry tomatoes. Of these compounds, the total flavonoid content was higher by 299.50 to 432.37 μg·g^−1^, and quercetin was the most abundant flavonoid in YT. Flavonoids are one of the main causes of color formation in yellow cherry tomatoes. The most abundant phenolic acid detected in the tomatoes was chlorogenic acid, which was the most abundant in RT and YT, followed by CT, and GT. RT and CT had the same total phenolic acid content, which was slightly lower than that of YT. In brief, YT had the highest content of phenolic compounds.

#### 3.3.5. PCA and CA of Phenolic Compounds

**Figure 7:**
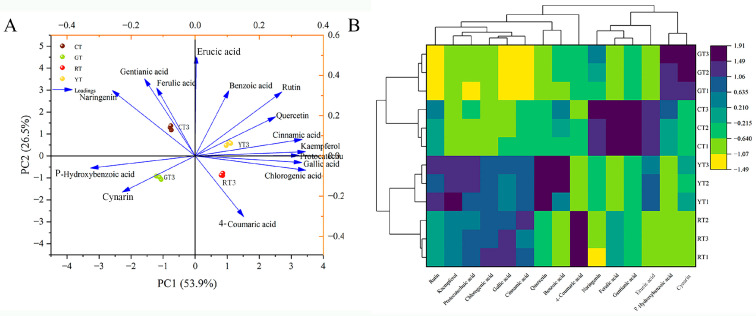
Principal component analysis (**A**) and cluster analysis (**B**) of phenolic compounds of different color cherry tomatoes.

The PCA-based classification model of phenolic compounds of differently colored cherry tomatoes is shown in Figure 7A. The two principal components extracted expressed 80% of the information, with PC1 and PC2 accounting for 53.9% and 26.5%, respectively. PC1 was positively correlated with nine phenolic compounds; in particular, cinnamic acid, kaempferol, protocatechuic acid, gallic acid and chlorogenic acid exhibited high loadings on PC1. Mustard acid, ferulic acid, gentianic acid and benzoic acid had high loadings on PC2. The four colored tomatoes were more separated on PC1 and PC2 and were in four different quadrants. YT scored high on both PC1 and PC2 with high phenolic content.

The hierarchical clustering heat map is shown in Figure 7B. The results of the hierarchical clustering were consistent with the results of the PCA. Little similarity was observed in the composition of phenolic acid compounds among the different colors of cherry tomatoes, and overall, YT had a higher phenolic acid content, especially for that of flavonoids.

#### 3.3.6. Electronic Nose Analysis

**Figure 8:**
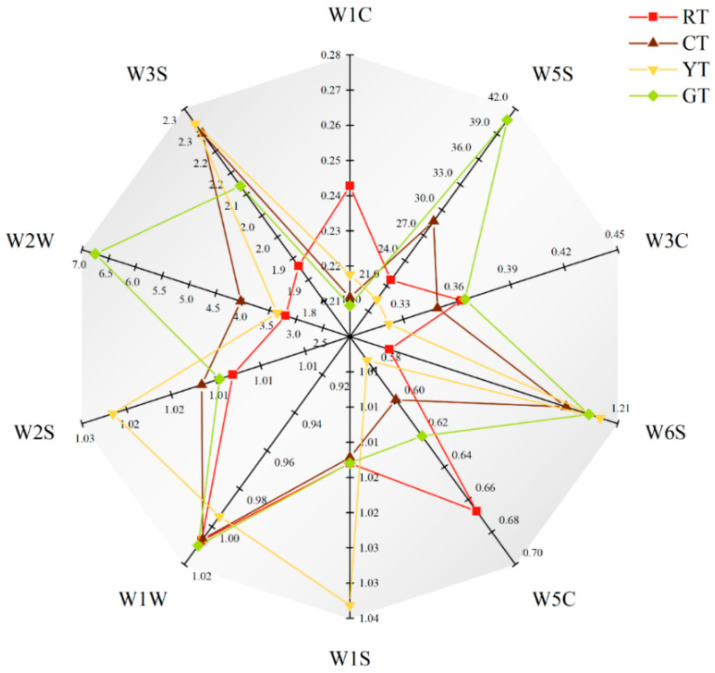
Electronic nose analysis of volatiles in differently colored tomatoes using the 10 sensors described in Table 1.

The radar map provided by the electronic nose analysis revealed the differences in the contribution values of differently colored tomatoes using 10 sensors. As shown in Figure 8, the W5S and W2W sensors had higher relative resistivity values among all tomatoes compared to the other sensors. Furthermore, the W2W, W5S and W1W sensors were sensitive to GT, indicating that GT have high nitrogen oxide and sulfide contents. The W3S, W2S, W1S, and W6S sensors were sensitive to YT, indicating that YT contained high levels of hydrides, methyl groups, alcohols, long-chain alkanes, aldehydes and ketones. In addition, the 10 electronic nose sensors were not sensitive to RT, and the volatile component level of RT was lower than that of the other tomatoes.

### 3.4. Comprehensive Evaluation of Flavor Qualities in Cherry Tomato Fruits of Different Colors

#### 3.4.1. Correlation Analysis of Flavor Indicators and Color Parameters

**Figure 9:**
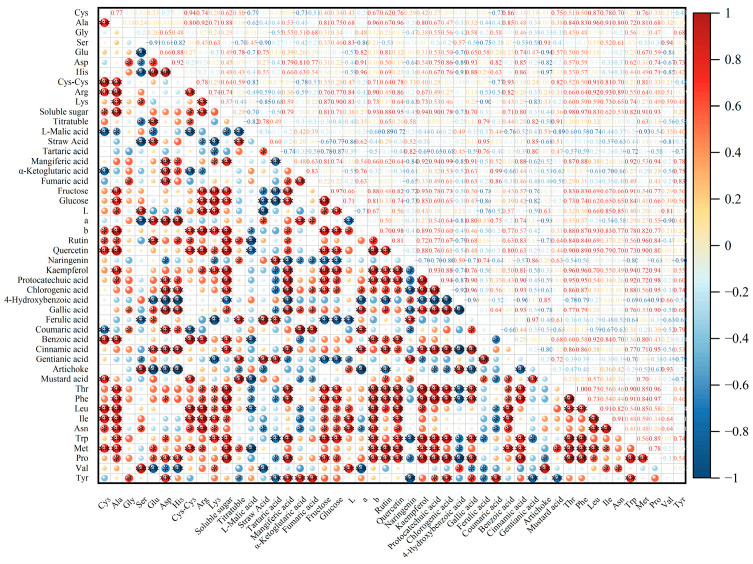
The heat map displays the Pearson correlation analysis. The values in the heat map are Pearson’s correlation coefficient. (*: *p* < 0.05, **: *p* < 0.01).

Pearson correlation analysis is often used to estimate the statistical associations between multiple variables [31]. The correlation between amino acids, soluble sugar, soluble solids, and organic acid content is shown in Figure 9. The heat map revealed a significant correlation between fructose, glucose and most amino acids (*p* < 0.05). Soluble solids were significantly highly and positively correlated with soluble sugars and Glu, Asp, His, Met, Pro, Phe and Thr. Tartaric acid exhibited a highly significant negative correlation with fructose and glucose levels. Amino acids play a crucial role in the distinctive tastes of vegetables, and the sweetness amino acids, Ala and Pro, were significantly positively correlated with soluble solids, soluble sugar, fructose and glucose. We noticed that Asp, the major sourness amino acid, had strong positive correlations with α-ketoglutaric acid. In addition, Glu, an amino acid responsible for sourness, was significantly correlated with oxalic acid. Furthermore, a correlation was observed between the color parameters and the flavor indicators. The L* value was highly positively and significantly correlated with cynarin, Ile, Asn, Val, Arg and Lys. The b* value was strongly and positively correlated with rutin, quercetin, and kaempferol; this value is an important measure of the difference between yellow and blue colors, suggesting that flavonoids are closely related to the formation of YT. Phenolic compounds were also significantly correlated with most amino acids. In brief, high correlation coefficient values were observed between the flavor quality indicators.

#### 3.4.2. PCA-Based Evaluation of Flavor Qualities in Cherry Tomato Fruits of Different Colors

To evaluate the flavor quality of differently colored tomato fruits accurately, a comprehensive analysis of 30 flavor indicators, using SPSS 22.0 to standardize the data, was performed. PCA was performed to reduce the dimensionality of the data, linearly transforming multiple variables into a few composite variables that reflect the original data in a simplified form. Three principal components were extracted based on eigenvalues greater than one. Furthermore, the cumulative variance contribution of the three principal components reached 86.62%, which was a good representation of all the main information of the test material, and could replace the original observations for the comprehensive evaluation of the flavor of tomatoes of different colors. As shown in Table 4, PC1 had the highest variance contribution (44.17%) and higher loadings for soluble sugar, soluble solids, fructose, glucose, Thr, Phe, Leu, Ile, Asn, Trp, Met, Pro, and Val in the feature vector. The variance contribution of PC2 was 23.34%, mainly associated with tartaric acid, citric acid, oxalic acid, fumaric acid, Glu, Asp and His. The variance contribution of PC3 was 19.11%, mainly associated with malic acid, α-ketoglutaric acid, Tyr, Gly and Ser.

After extracting the principal components, we computed the eigenvalues, eigenvectors, and standardized data to determine a score for each principal component, taking into account the variance contributed by each component. Finally, we calculated the total score for each tomato color. The flavor quality scores of differently colored tomatoes are shown in Table 5. A comprehensive analysis of 30 flavor indicators revealed a wide variation in flavor quality among differently colored tomatoes. As shown in Table 6, YT had the highest flavor quality, with the four tomato groups ranking as YT > RT > CT > GT. Specifically, YT demonstrated superior performance in terms of glycolic acid and amino acid content.

## 4. Discussion

Cherry tomatoes are important vegetables, rich in nutrients and bioactive compounds and renowned for their flavor, texture, and color [32,33]. The colors of the skin and flesh together determine the appearance of cherry tomatoes, and the source of these colors is mainly the accumulation of pigments, the main determinants of which are chlorophyll, carotenoids and flavonoids [34]. The ripening process of tomato fruits is accompanied by the transformation of chloroplasts into chromoplasts, and the biosynthesis and accumulation of carotenoids in the chromoplasts of fruit cells [35]. The presence of chlorophyll makes young tomato fruit predominantly green and functions as a photosynthetic mechanism [36]. The yellow, orange, and red colors of fruits are due to their carotenoids and flavonoid contents, and these pigments can influence fruit quality [37]. Chlorophyll is essential for photosynthesis, enabling plants to capture solar energy and producing carbohydrates [38], Powell et al. [39] state that the GLK expression, which regulates chloroplast development, affects the accumulation of sugars and carotenoids in green tomato fruit. Flavonoids have antioxidant properties [40], and are precursors for the synthesis of flavor substances [41]. Variations in the type and content of these pigments not only result in different fruit colors, but also play a role in the nutritional and flavor value of the fruit. Our results showed that lycopene accumulation was a major factor in the reddening of tomato fruits and was detected in RT and CT, but not in GT or YT. The color parameters of the four fruit colors of cherry tomatoes differed significantly, reflecting a difference in pigment accumulation to some extent [42]. The L* value reflects brightness, the a* value indicates colors ranging from green to red (positive values reflect a red bias, negative values reflect a green bias), and the b* value indicates colors ranging from blue to yellow (positive values reflect a yellow bias, negative values reflect a blue bias) [43]. Our analyses revealed that RT has the largest a* value, GT has the smallest a* value and YT has the largest b* value. Therefore, pigment accumulation is one of the main factors influencing color parameters. Firmness is an important indicators of fruit maturity and quality during storage. Our results indicate that at the same maturity level, CT fruit cell walls contained more proto-cellulose and were resistant to storage. The horizontal and vertical diameters and fruit shape index reflect fruit shape, which varies from color to color.

Cherry tomatoes are rich in many healthy compounds, including lycopene, vitamin C, and minerals, which are good dietary antioxidants [44]. Vitamin C is an important antioxidant that neutralizes superoxide and hydroxyl radicals. Lycopene is the most abundant carotenoid in tomato fruits and an important natural food colorant with strong antioxidant properties; it not only acts as a pigment that affects the color of the tomato fruit but is also an important bioactive component [45]. In the present study, lycopene was not detected in green and yellow tomatoes, but their vitamin C content was high, which is consistent with the results of Chang et al. [33]. The lycopene content of RT tomatoes was the highest, reaching 48.08 mg kg^−1^, followed by CT with 34.26 mg kg^−1^, which was 47.37% lower than that of RT. In addition, the soluble protein of green and yellow tomatoes was significantly higher than that of other tomatoes. RT and CT also had a larger single fruit weight, possibly attributed to the prioritization of yield over nutritional quality for RT during the domestication process. In addition, consistent cultivation environments and management practices likely contributed to the minimal differences in the mineral element content across tomato colors.

Amino acid composition is one of the most important indicators of the nutritional value and flavor of tomato fruits [46], and is not only involved in protein biosynthesis but is also significantly associated with human taste perception [47]. Some amino acids are defined as taste-presenting amino acids [48], such as sweetness amino acids including Ala, Gly and Ser, and sourness amino acids including Asp and Glu [49]. Our results indicated that cherry tomatoes exhibit relatively high levels of Thr, Phe, Trp, Lys, Leu, Cys, Ala, Arg and Pro. RT had high Asp, His and Gly contents. GT had high Val and Ser contents and CT had lower amino acid contents than the other colors. YT were the richest in amino acids, such as Ala, Pro, Leu, Trp and Phe, of which Trp and Phe are aromatic amino acids involved in the shikimic acid pathway and play an important role in the aroma formation of fruits [50,51]. These results suggest that amino acids are also involved in the synthesis of flavor substances, thereby enriching the flavor hierarchy in foods.

The amino acid content of the four colored cherry tomatoes varied, which also allowed for significant differences in the type and content of volatiles [52]. Chang et al. analyzed the volatile components of four differently colored cherry tomatoes and found that the type and content of volatile components varied greatly between varieties, especially for volatiles synthesized with carotenoids as precursors [53]. In our study, the volatile substances with high contents in cherry tomatoes were nitrogen oxides and organic sulfides. Furthermore, YT contained high levels of aldehydes, ketones, and alcohols; these three volatile categories are the main substances that make up the main flavor of tomatoes. Researchers have identified more than 400 volatiles in tomatoes and screened more than 30 characteristic volatiles based on odor thresholds [54]. However, this study did not allow for the qualitative analysis of all volatiles. A more comprehensive analysis of the volatiles in cherry tomatoes of different colors should be conducted in future studies.

Soluble solids, sugar, and organic acid contents have an important influence on the taste and flavor of cherry tomatoes and are important indicator of their quality [55]. Soluble solids is the general term for all compounds dissolved in water in foods, including sugars, acids, vitamins, minerals, etc. [56]; it is a comprehensive indicator of the flavor of the fruit. YT had a high level of soluble solids, which is consistent with the results of our individual component tests. In our study, the soluble sugar of YT was also significantly higher than that of other tomatoes, and RT had the second highest soluble sugar content, albeit lower at 11.11%. Six notable organic acid fractions were identified in this study. Citric and malic acids are the predominant organic acid components in cherry tomatoes [57], while fumaric acid, oxalic acid, tartaric acid and α-ketoglutaric acid are present in lower levels. Different ratios of sugar and acid content contribute to the different flavors of cherry tomatoes of different colors. Yellow tomatoes with a higher sugar content and lower acid content are more acceptable to the general public, which means that YT taste the best. This may be because the YT is closer to the wild variety, while the breeding of the commonly cultivated RT prioritized yield over relevant indicators such as nutrition and flavor during the domestication process.

Phenolic acids and flavonoids, the main antioxidant in tomato fruits, have high nutritional and health-promoting properties [40]. Our results showed that the phenolic acid and flavonoid content of YT were higher, reaching 470.70 μg·g^−1^, which was 3.9% higher than that of RT, which was consistent with the fact that flavonoid was the main reason for the yellow formation of the tomato [58]. A total of 4 flavonoids and 11 phenolic acids were detected, with YT having significantly higher rutin, quercetin, kaempferol, protocatechuic acid, chlorogenic acid, benzoic acid and cinnamic acid contents than the other colored cherry tomatoes. The accumulation of flavonoids and phenolic acids also improved the quality of the YT.

PCA can recombine original variables into a new set of mutually uncorrelated composite variables; as a rule, the selected variables should cover more than 80% of the total information in the original data [59]. Previously, Cao et al. [60] performed a comprehensive assessment and screening of tomatoes for cold tolerance using PCA, and Patras et al. [61] applied PCA to classify fruits and vegetables. Shi et al. [62] used PCA to comprehensively evaluate the nutrient composition and quality traits of dried jujube fruits from seven production areas. In our study, the evaluation of the flavor of four colored tomatoes, based on PCA, suggested that the flavor ranking was YT > RT > CT > GT.

## 5. Conclusions

In this study, the analysis and identification of primary and secondary metabolites in four differently colored cherry tomatoes, namely Song of Youth (YT), Sweet (RT), Chocolate (CT), and Romeo (GT), was conducted using targeted metabolism techniques. The findings revealed that YTs have higher amino acid, phenolic acid, and soluble sugar contents compared to other colors, GT have higher vitamin C and soluble protein contents, and RT and CT have higher lycopene contents. Furthermore, cherry tomato flavor formation was positively correlated with amino acid content. A comprehensive evaluation of flavor quality based on PCA showed that the top flavor ranking was given to YT, followed by RT, CT, and GT. Our work provides a starting point for future investigations into the mechanisms behind color and flavor formation in cherry tomatoes.

## Figures and Tables

**Table 1:** Ten chemical sensing elements of electronic nose.

Serial Number	Sensor Number	Substance Types	Sensor Performance Description
1	W1C	Aromatic	Aromatic components and benzenes
2	W5S	Broadrange	High sensitivity, sensitive to nitrogen oxides
3	W3C	Aromatic	Sensitive aromatic components and ammonia
4	W6S	Hydrogen	Mainly selective to hydride
5	W5C	Arom-aliph	Aromatic components of short-chain alkanes
6	W1S	Broad-methane	Sensitive to methyl groups
7	W1W	Sulfur-organic	Sensitive to sulfides
8	W2S	Broad-alcohol	Sensitive to alcohols, aldehydes and ketones
9	W2W	Sulph-chlor	Aromatic components and sensitive to organic sulfides
10	W3S	Methane-aliph	Sensitive to long-chain alkanes

**Table 2:** The firmness, horizontal diameter, vertical diameter, single fruit weight, and fruit shape index of different color tomatoes.

Color	Firmness (kg·cm^−2^)	Horizontal Diameter (cm)	Vertical Diameter (cm)	Single Fruit Weight (g)	Fruit Shape Index
RT	2.48 ± 0.10 b	3.05 ± 0.17 a	2.97 ± 0.0.2 b	31.16 ± 1.39 a	0.98 ± 0.07 b
CT	3.63 ± 0.16 a	3.00 ± 0.15 a	3.83 ± 0.07 a	26.68 ± 1.09 b	1.28 ± 0.04 a
YT	2.54 ± 0.16 b	2.90 ± 0.01 a	2.88 ± 0.10 b	21.27 ± 0.43 c	0.99 ± 0.04 b
GT	2.26 ± 0.03 b	2.92 ± 0.06 a	3.88 ± 0.13 a	20.53 ± 0.79 c	1.33 ± 0.07 a

Values are presented as the mean ± standard deviation (*n* = 3), and the different letters signify significant differences between the values within the same column (*p* < 0.05).

**Table 3:** The amino acid contents of differently colored tomatoes.

Amino Acids (mg/100 g)	Color			
	RT	CT	YT	GT
Essential amino acids (EAAs)				
Thr ^C^	70.26 ± 0.52 b	55.86 ± 0.14 c	87.71 ± 1.28 a	49.75 ± 3.13 d
Lys ^D^	144.94 ± 2.41 c	121.83 ± 3.91 d	170.87 ± 3.15 a	154.85 ± 3.37 b
Trp ^A^	20.41 ± 0.68 b	13.47 ± 0.15 d	22.70 ± 0.82 a	15.80 ± 0.79 c
Phe ^A^	67.18 ± 0.05 b	52.86 ± 0.40 c	82.53 ± 1.19 a	47.21 ± 3.23 d
Val ^B^	5.38 ± 0.12 c	5.83 ± 0.09 c	7.84 ± 0.19 b	9.48 ± 0.60 a
Leu ^B^	6.96 ± 0.13 d	10.70 ± 0.07 b	21.20 ± 0.28 a	8.96 ± 0.37 c
Ile ^B^	13.22 ± 0.30 d	14.63 ± 0.11 c	19.22 ± 0.31 a	16.03 ± 0.67 b
Met ^B^	6.37 ± 0.17 c	7.74 ± 0.06 b	11.31 ± 0.36 a	3.47 ± 0.17 d
Non-essential amino acids (NEAAs)				
Ser ^C^	22.84 ± 0.57 c	21.05 ± 1.95 c	33.39 ± 0.35 b	43.35 ± 0.22 a
Tyr ^A^	9.64 ± 0.25 a	4.14 ± 0.11 c	7.22 ± 0.26 b	6.74 ± 0.45 b
Cys-Cys ^A^	0.37 ± 0.01 d	0.63 ± 0.01 b	0.77 ± 0.01 a	0.47 ± 0.03 c
Cys	0.87 ± 0.06 c	1.36 ± 0.08 b	1.67 ± 0.06 a	1.07 ± 0.11 c
Arg ^B^	20.10 ± 2.03 c	22.06 ± 0.46 c	38.16 ± 1.40 a	26.11 ± 0.63 b
His ^C^	315.68 ± 3.74 a	248.71 ± 3.67 b	250.61 ± 5.31 b	145.72 ± 2.53 c
Pro ^C^	13.26 ± 0.24 b	5.56 ± 0.08 c	16.57 ± 0.35 a	2.40 ± 0.11 d
Gly ^C^	3.23 ± 0.15 a	2.59 ± 0.15 b	2.89 ± 0.21 ab	2.67 ± 0.29 ab
Ala ^C^	20.59 ± 0.35 b	20.90 ± 0.05 b	34.53 ± 1.29 a	21.30 ± 1.46 b
Glu ^D^	1806.77 ± 34.49 a	1892.54 ± 14.10 a	1666.38 ± 43.18 b	747.35 ± 42.17 c
Asp ^D^	940.42 ± 14.03 a	464.91 ± 8.58 c	601.98 ± 21.05 b	293.44 ± 26.00 d
Asn ^C^	13.43 ± 0.48 c	14.33 ± 0.54 bc	17.72 ± 0.31 a	15.78 ± 0.60 b
Total EAAs	334.76 ± 3.31 b	282.92 ± 4.71 d	423.38 ± 3.15 a	305.54 ± 11.72 c
Total NEAAs	3167.24 ± 50.41 a	2698.78 ± 24.18 b	2671.89 ± 66.98 b	1306.40 ± 73.16 c
Total A	97.62 ± 0.96 b	71.10 ± 0.53 c	113.21 ± 2.19 a	70.22 ± 4.47 c
Total B	52.06 ± 1.33 c	60.97 ± 0.55 b	97.73 ± 2.48 a	64.05 ± 2.43 b
Total C	459.30 ± 5.03 a	369.00 ± 5.69 b	443.43 ± 5.93 a	280.96 ± 7.11 c
Total D	2892.14 ± 48.45 a	2479.28 ± 25.35 b	2439.23 ± 62.10 b	1195.64 ± 70.45 c
Total amino acids	3501.00 ± 52.11 a	2981.71 ± 28.61 b	3095.27 ± 69.08 b	1611.94 ± 84.31 c

Values are given as the mean ± standard deviation (*n* = 3), and the different letters within each column were significantly different (*p* < 0.05). “^A^”: Aromatic amino acid; “^B^”: Bitterness amino acid; “^C^”: Sweetness amino acid; “^D^”: Sourness and umami amino acid.

**Table 4:** Flavonoids and phenolic acid contents of differently colored tomatoes.

Phenolic Components (μg·g^−1^ DW)	Color			
	RT	CT	YT	GT
**Flavonoids**				
Rutin	77.356 ± 1.95 b	83.122467 ± 1.90 b	127.1793 ± 3.50 a	5.1879 ± 0.18 c
Quercetin	79.6947 ± 1.34 b	70.3163 ± 0.72 b	342.2132 ± 7.73 a	28.9946 ± 0.70 c
Naringenin	8.3033 ± 0.56 d	34.7853 ± 0.43 a	14.2285 ± 1.24 c	20.8541 ± 2.59 b
Kaempferol	2.7696 ± 0.07 b	0.4819 ± 0.01 d	4.5938 ± 0.14 a	0.8037 ± 0.07 c
Total flavonoids	168.1236 ± 2.41 b	188.7059 ± 2.25 b	488.2148 ± 12.00 a	55.8402 ± 2.53 c
**Phenolic acids**				
Protocatechuic acid	10.5708 ± 0.21 b	4.9250 ± 0.35 c	12.2806 ± 0.90 a	3.7167 ± 0.27 c
Chlorogenic acid	259.3327 ± 1.74 a	188.7604 ± 1.02 b	253.2697 ± 3.08 a	183.2061 ± 0.66 b
P-hydroxybenzoic acid	4.1595 ± 0.08 c	10.3902 ± 0.79 b	5.6864 ± 0.15 c	15.3562 ± 0.90 a
Gallic acid	103.1685 ± 2.48 a	66.8421 ± 2.29 c	91.4512 ± 1.18 b	53.9213 ± 1.08 d
Ferulic acid	2.7835 ± 0.18 b	10.5343 ± 0.25 a	ND	ND
4-Coumaric acid	5.1617 ± 0.10 a	0.41193 ± 0.05 c	0.3222 ± 0.03 c	0.9337 ± 0.07 b
Benzoic acid	65.5487 ± 0.39 c	82.0720 ± 3.85 b	100.2444 ± 5.83 a	72.4851 ± 1.97 bc
Cinnamic acid	0.3800 ± 0.04 a	0.1680 ± 0.01 b	0.3746 ± 0.01 a	ND
Gentianic acid	1.9084 ± 0.05 b	82.5283 ± 4.17 a	ND	ND
Cynarin	ND	1.1651 ± 0.13 c	1.9026 ± 0.15 b	5.4345 ± 0.24 a
Erucic acid	ND	6.9455 ± 0.05 a	5.1669 ± 0.22 b	ND
Total phenolic acid	453.0138 ± 2.12 b	454.7428 ± 2.27 b	470.6987 ± 4.16 a	335.0535 ± 2.04 c

Values are presented as the mean ± standard deviation (*n* = 3), and the different letters signify significant differences between the values within the same row (*p* < 0.05). ND: Not Detected.

**Table 5:** Eigenvalue and accumulative contribution rate of tomato fruit flavor quality.

Component Number	Eigenvalue	Contribution Rate	Cumulative %
1	13.25	44.17	44.17
2	7.00	23.34	67.51
3	5.73	19.11	86.62

**Table 6:** Combined flavor quality scores for differently colored tomatoes.

Color	Total Score	Rankings
RT	1.93	2
CT	−1.04	3
YT	2.49	1
GT	−1.36	4

## Data Availability

The original contributions presented in the study are included in the article, further inquiries can be directed to the corresponding author.
